# Structure and Molecular Dynamics Simulations of Protein Tyrosine Phosphatase Non-Receptor 12 Provide Insights into the Catalytic Mechanism of the Enzyme

**DOI:** 10.3390/ijms19010060

**Published:** 2017-12-26

**Authors:** Hui Dong, Francesco Zonta, Shanshan Wang, Ke Song, Xin He, Miaomiao He, Yan Nie, Sheng Li

**Affiliations:** 1 Key Laboratory of Tianjin Radiation and Molecular Nuclear Medicine, Institute of Radiation Medicine, Chinese Academy of Medical Sciences & Peking Union Medical College, Tianjin 300192, China; hexin@irm-cams.ac.cn; 2 Shanghai Institute for Advanced Immunochemical Studies, ShanghaiTech University, Shanghai 201210, China; fzonta@shanghaitech.edu.cn (F.Z.); wangss2@shanghaitech.edu.cn (S.W.); Songk@shanghaitech.edu.cn (K.S.); hemm@shanghaitech.edu.cn (M.H.); 3 University of Chinese Academy of Sciences, Beijing 100049, China; 4 Institute of Biochemistry and Cell Biology, Shanghai Institutes for Biological Sciences, Chinese Academy of Sciences, Shanghai 200031, China

**Keywords:** PTPN12, molecular dynamics, structure

## Abstract

Protein tyrosine phosphatase non-receptor 12 (PTPN12) is an important protein tyrosine phosphatase involved in regulating cell adhesion and migration as well as tumorigenesis. Here, we solved a crystal structure of the native PTPN12 catalytic domain with the catalytic cysteine (residue 231) in dual conformation (phosphorylated and unphosphorylated). Combined with molecular dynamics simulation data, we concluded that those two conformations represent different states of the protein which are realized during the dephosphorylation reaction. Together with docking and mutagenesis data, our results provide a molecular basis for understanding the catalytic mechanism of PTPN12 and its role in tumorigenesis.

## 1. Introduction

Protein tyrosine phosphorylation, which is precisely balanced by the activities of protein tyrosine kinases (PTKs) and phosphatases (PTPs), plays a critical regulatory role in most aspects of cell functions, such as metabolism, cell growth, differentiation, migration, and apoptosis [1,2]. Dysregulation of this balance results in various abnormities, including cancer, metabolic diseases, and immunological diseases [3,4,5]. PTPs have emerged as promising therapeutic targets and potent and selective inhibitors were successfully developed [6,7]. Structure-based rational approaches have been successful in the development of PTP inhibitors [8].

Protein tyrosine phosphatase non-receptor 12 (PTPN12), which belongs to the classical PTPs, was initially identified as a non-transmembrane PTP with C-terminal segment rich in Pro, Glu, Asp, and Ser (P-E-S-T, PTP-PEST) [9]. The non-transmembrane 4 (NT4) subfamily of PEST domain-containing PTPs also includes BDP1/PTPN18 and Lyp/PTPN22 [10]. PTPN12 is expressed ubiquitously in a wide variety of tissues and is thought to be a critical regulator of cell adhesion and migration, correlating with the activities of its substrates such as p130cas, focal adhesion associated-kinase (FAK), pyruvate kinase (Pyk2), and proline-serine-threonine phosphatase-interacting protein (PSTPIP) [10]. Analysis of PTPN12-knockout embryos revealed associated defects in somatogenesis, vasculogenesis, and liver development [11].

Several PTPs have been extensively studied and identified as tumor-suppressor genes, such as phosphatase and tensin homolog (PTEN) [12,13], protein tyrosine phosphatase kappa (PTPRK) [14], and Fas-associated phosphatase 1 (FAP-1) [15]. More recently, Tingting et al. reported that the loss of PTPN12 activity by deletions, mutations, and loss of expression result in aberrant activation of the human epidermal growth factor receptor 2 (HER2)/epidermal growth factor receptor (EGFR)-centered receptor tyrosine kinase-signaling network, which involves more than 60 proteins, in triple negative breast cancer (TNBC) [16]. Several mutations were identified in the catalytic domain. Restoring PTPN12 expression in breast cancer cells exhibiting PTPN12 deficiency suppresses proliferation, tumorigenesis, and metastasis.

The tertiary structure of the ~280-residue PTP catalytic domain is highly conserved among family members [17,18,19]. Indeed, they all share a common overall folding composed of a central β-sheet with two and six α-helices on each side [18]. The PTP signature motif or P-loop (HCX5R) comprises residues at the base of the active site cleft, forming the phosphate-binding loop. The cysteine in the P-loop acts as a nucleophile and transiently accepts phosphate ions during catalysis [20]. The tryptophan-proline-aspartate (WPD) loop is flexible; it moves several angstroms to form a closed conformation, and it traps the phosphotyrosine upon substrate binding [21,22]. Other motifs include the catalytic water motif (Q-loop), which is involved in the second hydrolysis step of the phosphocysteine-enzyme complex [14,23], and the phosphotyrosine-recognition loop [19,21,24,25].

Here, we report the crystal structure of the wild type PTPN12 catalytic domain refined to 1.62 Å resolution. Crystallographic data show that the catalytic cysteine (Cys231) can adopt two conformations in this structure, and to interpret this evidence, we built an atomistic model based on molecular dynamics simulations. Our results suggest that one configuration corresponds to a transient state of the enzyme, in which Cys231 is covalently bound to a phosphate ion, while the other corresponds to the phosphate free form of the enzyme. Finally, we provide evidence that some mutations in the catalytic domain, which are found in cancer, can inactivate the enzyme and thus might be involved in tumorigenesis.

## 2. Results

### 2.1. Overall Structure of PTPN12

**Figure 1:**
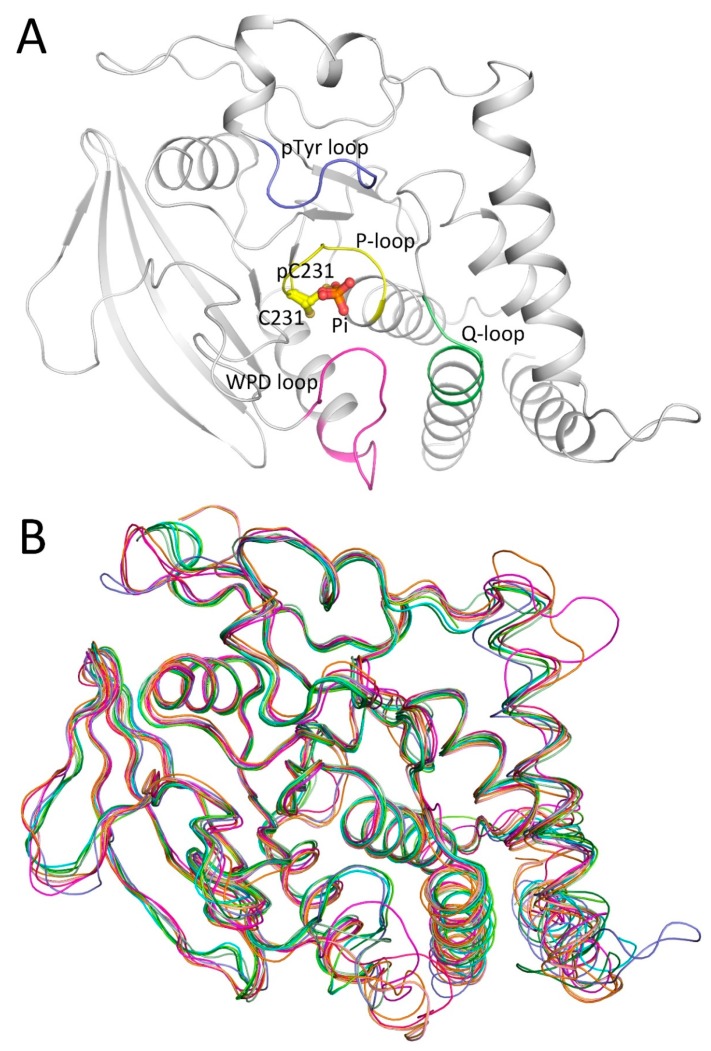
Structure of the protein tyrosine phosphatase non-receptor 12 (PTPN12) catalytic domain. (**A**) Overall structure of the PTPN12 catalytic domain. The PTPN12 catalytic domain, pTyr recognition loop, P-loop, tryptophan-proline-aspartate (WPD)loop, and Q-loop are colored grey, blue, yellow, magenta, and green, respectively. The free phosphate ion and two conformations of catalytic cysteine 231 (pC231 and C231) are shown in stick-ball models; (**B**) comparison of the structures of the protein tyrosine phosphatase (PTP) catalytic domain. Blue, PTPN12; light green, free PTPN18, PDB ID 2OC3; **green**, PTPN18 in complex with the human epidermal growth factor receptor 2 (HER2)pY1248 peptide, Protein Data Bank (PDB) ID 4GFU; **dark green**, PTPN18 with HER2 pY1196, PDB ID 4GFV; **cyan**, PTPN18 with HER2 pY1112, 4NND; **pink**, free PTPN22 catalytic domain, 2P6X; **magenta**, free PTPN22, 2QCJ; **red**, PTPN22 with inhibitor I-C11, 2QCT; **orange**, PTPN22 with phosphate, 3H2X; **yellow**, PTPN22 with inhibitor L75N04, 4J51. Figures were prepared using PyMOL (DeLano Scientific LLC, South San Francisco, CA, USA).

The structure of the PTPN12 catalytic domain was solved by molecular replacement using the structure of a Lyp catalytic domain (Protein Data Bank (PDB) ID 2QCJ) as a search model and refined to 1.62 Å resolution. The overall structure of the PTPN12 catalytic domain was found to be similar to the NT4 subfamily of PTPs (PDB IDs 2OC3, 2P6X, 2QCJ, 2QCT, 3H2X, 4GFU, 4GFV, 4J51, and 4NND) [17,26,27,28,29] with a RMSD of Cα atoms less than 1.4 Å, respectively (Figure 1A,B). The PTPN12 catalytic domain adopted a canonical α/β folded structure typical of a central eight-stranded β-sheet, with six and two α-helices on each side.

**Figure 2:**
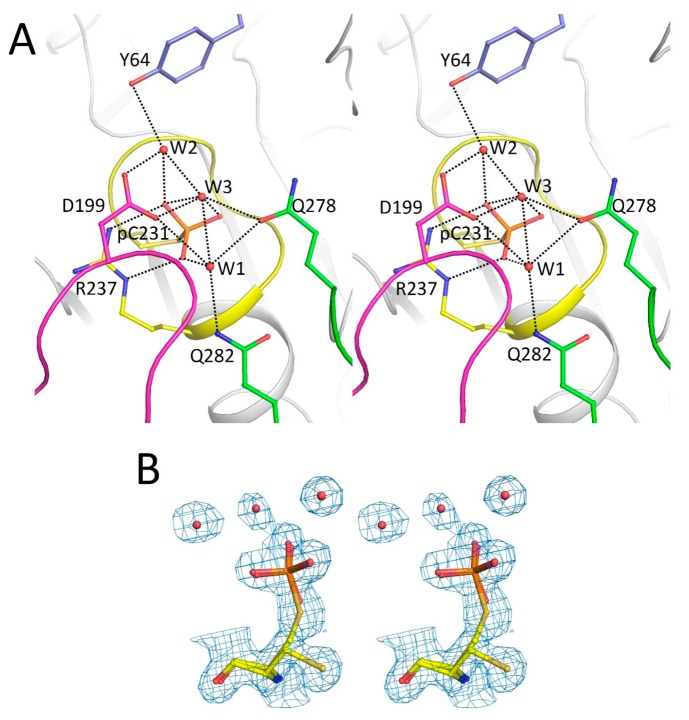
Catalytic site of PTPN12. (**A**) A stereoview showing the hydrogen-bonding interactions between water molecules and residues in the catalytic site. The coloring is the same as that used in Figure 1. Hydrogen bonds are indicated by dashed lines; (**B**) a representative composite-omit-map (1.0 contour level) of the catalytic Cys231 residue and surrounding water molecules. Figures were prepared using PyMOL (DeLano Scientific LLC, South San Francisco, CA, USA).

The PTP signature motif (P-loop) was composed of residues 230–237 (Figure 1A). The catalytic Cys231 adopted dual conformations, one of which was assigned as the phosphoryl–cysteine intermediate [23], coupled with three water molecules (discussed below) (Figure 2A,B). The WPD loop, which adopted a closed conformation, was well defined as residues 197–208. Other structural motifs include the pTyr recognition loop (residues 58–64) and the Q-loop (residues 278–285) [19] (Figure 1A). Some minor structural differences were observed between PTPN12 and other NT4 PTPs. Spatial displacements were observed in the regions around Thr81–Asp85 and Pro257–Phe260, compared with the PTPN18 and PTPN22 structures. A 5-residue insertion, which does not exist in PTPN18 and PTPN22, formed a loop between the α1 and α2 helices. In addition, residues Ser39–Lys46 of PTPN12 composed part of the α2 helix, which is similar to PTPN18, but not PTPN22 [26].

### 2.2. Catalytic Cys231 and Water Molecules

During the early stages of the structure refinement, a clear tetrahedral-shaped density was observed at the binding site of the tyrosyl–phosphate, which was very close to the catalytic Cys231. As phosphate ions were present in the crystallization buffer, the density was assigned as a phosphate ion during the early stages of refinement. However, after several rounds of model building and refinement, the distance between the Cys231 Sγ and oxygen atom of the phosphate ion (1.3 Å) appeared to be too short. Moreover, the catalytic Cys231 adopted dual conformation. By assuming that the PTPN12 underwent a slow reaction during the protein production or crystallization procedure, Cys231 was modeled as an alternate conformation of phosphoryl–cysteine with occupancy of 0.7, and the other as an alternate conformation of unphosphorylated cysteine with occupancy of 0.3. The phosphoryl–cysteine conformation can be considered a phosphoryl–enzyme intermediate. The phosphoryl moiety interacted with the side chain of Arg237 (Arg235 in PTPN18, Arg233 in PTPN22, or Arg221 in PTP1B) and the backbone amides of the P-loop (Figure 2A). The non-phosphorylated conformation of Cys231 adopted a different side-chain orientation with a relative rotation of ~100°.

**Figure 3:**
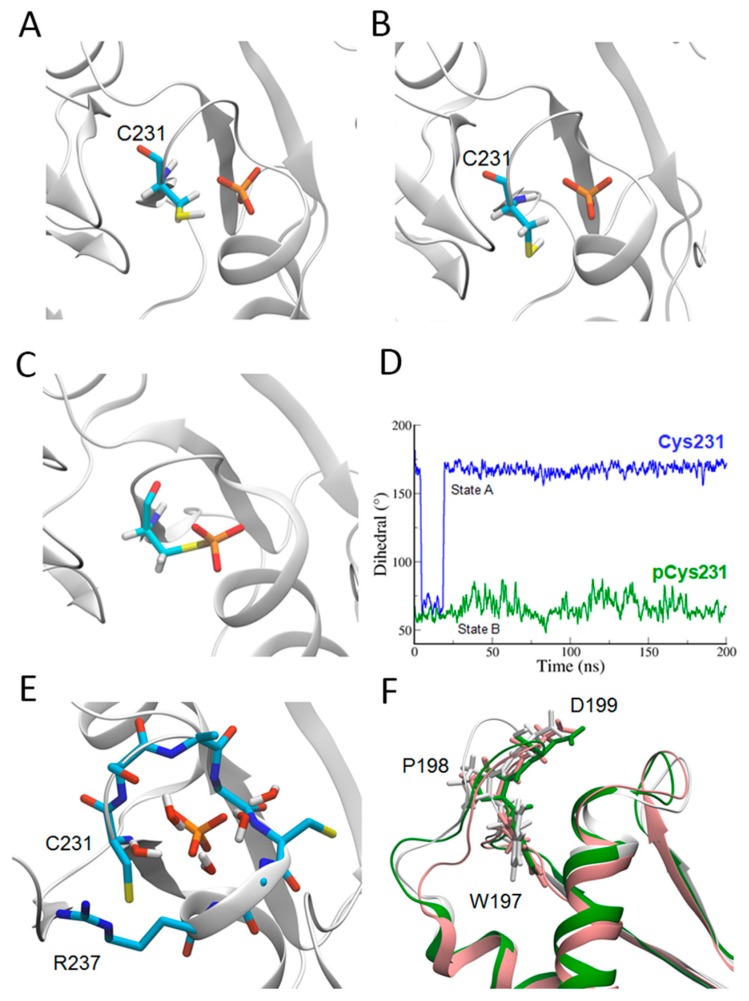
PTPN12 in molecular dynamics (MD) simulations. Panels (**A**,**B**) show two snapshots of two different states of C231 along the MD trajectory, which correspond to configurations in which the dihedral angles between the main chain and the side chain of the cysteine are, respectively, 70° or 170°. In the presence of the phosphoryl–cysteine, no transition was observed, as shown in panel (**C**), and graphically in panel (**D**); Panel (**E**) shows the positions of water molecules in the MD simulation. The spatial distribution of the water molecules in the simulation was slightly different due to the conformational change initiated by the bond breaking between C231 and the phosphate ion. Panel (**F**) shows the comparison between the loop containing the WPD motif in the crystal structure (**green**), in the MD model in the presence (**white**) or absence of the phosphate ion (**pink**). Figure 3A–C,E,F were prepared using PyMOL(DeLano Scientific LLC, South San Francisco, CA, USA). Figure 3D was prepared using Gromacs 4.6.7 package (version 4.6.7, University of Groningen, Holland, The Netherlands).

In order to verify our hypothesis and highlight the mobility of relevant residues and structural elements, we performed a series of molecular dynamics (MD) simulations, with or without the covalent bound between the phosphate ion and the cysteine. In the first case, Cys231 was parameterized as a standard cysteine, while in the second case we derived the force field parameters of the phosphoryl–cysteine by using the method as in ref [30]. MD simulations showed that two different orientations of the side chain of Cys231 are possible, in agreement with the two different conformations found in the crystal structure. In particular, the hydrogen atom, which is bonded to the sulfur atom of a standard cysteine side chain, slightly superimposed with the phosphate causing the reorientation of Cys321 side-chain dihedral angle by approximately 100°, similarly to what we observed in the alternate orientation of the crystal structure (Figure 3A,B), while in the presence of the phosphoryl–cysteine we do not observe any transition (Figure 3C). The alternative configuration, which we observe in the absence of the covalent bound between the phosphate ion and Cys231, stabilizes after the first 40 ns of dynamics, and is overall realized for approximately 90% of the corresponding MD trajectory (Figure 3D), indicating the possibility that the two orientations of Cys231 side chains correspond to two different chemical states of the complex phosphate–cysteines. Moreover, this configuration change, which in our simulation is triggered by the protonation of Cys231 side chain, could be necessary for initiating the process of release of the phosphate ion. Considering the results of the MD simulations, an extra isolated phosphate ion was defined at the position of the phosphoryl moiety of the phosphoryl–cysteine with an optimized occupancy of 0.12 (Figure 1B and Figure 2A). The interactions of this phosphate ion with the surrounding residues were almost identical to those of the cysteinyl–phosphate.

Clear densities were observed for three water molecules interacting with the thiophosphate group in the crystal structure (Figure 2B), similar to the phosphocysteine–PTP1B structure (PDB ID 1A5Y) reported previously [23]. Among them, the W2 water molecule is ideally positioned for nucleophilic attack on the cysteinyl–phosphate intermediate, thereby forming hydrogen bonds with an oxygen atom of the cysteinyl–phosphate, the side chains of Tyr64 and Asp199, and W3 (Figure 2A). The W1 water molecule forms hydrogen bonds with the cysteinyl–phosphate, Asp199, Gln278, Gln282, and backbone amide of the P-loop. The W3 water molecule interacts with the cysteinyl–phosphate, Asp199, Gln278, and water molecules W1 and W2 (Figure 2A).

As in the crystal structure, water molecules were present around the phosphate ion in the MD simulation. The simulation clearly indicated a dynamic equilibrium, wherein new water molecules replaced those that diffused away. The reorientation of the Cys231 side chain created a pocket for the water molecule (Figure 3E). This water molecule was well positioned to push Arg237 away from the active site and thus could contribute to the release of the phosphate.

We compared the PTPN12 structure with the structures of other NT4 phosphatases. The side chain of the catalytic Cys227 residue of a substrate-free PTPN22 structure (PDB ID 2P6X) pointed to the center of the P-loop in a previous study [17], in the same orientation as the phosphoryl–cysteine in our structure. Moreover, the catalytic cysteines in previously described phosphoryl–peptide–bound PTPN18 structures (PDB IDs 4GFU, 4GFV, and 4NND) [28] assumed the same orientation as did the phosphoryl–cysteine in our structure. Those data indicated that Cys231 was in the same orientation immediately before and after its phosphorylation. Considering the surrounding residues, the conformation of Arg237 of PTPN12 was identical to Arg235 of phosphoryl–peptide–bound PTPN18, and Arg221 of phosphoryl–cysteine–PTP1B. In addition, a disulfide bond was observed between Cys227 and Cys129 of PTPN22 in the reduced state [27]. It was proposed that such a disulfide bond can help stabilize the conformation of the WPD loop. In our PTPN12 structure, such a disulfide bond was not observed.

Considering all of the above observations, we suggest that the conformation of the PTPN12 catalytic domain with the phosphoryl–cysteine represented the state intermediately after the phosphate ion was transferred from the substrate to the enzyme, while the other unphosphorylated conformation was the state after the phosphate ion was released from the enzyme.

### 2.3. WPD Loop

The WPD loop in the PTPN12 structure was well defined as a closed conformation, which is very similar to those of PTPN18 in complex with phosphoryl–peptide (PDB IDs 4GFU, 4GFV, and 4NND) [28]. Only Ser204 and 205 (Ser202 and 203 in PTPN18) undergo approximately 3.5 Å displacement in the comparison. Those two residues are far from the reaction center (17 Å away from the cysteinyl–phosphate). The interaction between Trp197 and Arg237 was broken in the closed WPD loop, as in previous reports [19,27].

In the MD simulation, the position of the WPD loop fluctuated in the presence of the phosphate ion, and the loop reached a configuration (Figure 3F, white color) that was significantly different from that of the crystal structure (Figure 3F, green color).

During the simulation, the phosphate ion was strongly stabilized by the backbone of the residue in the P-loop. In contrast, neither the WPD loop nor the side chain of Arg237 seemed to stably interact with the ion. The former lost its interaction with the phosphate at the very beginning of the simulation (after 500 ps), while the change in its configuration created access to the ion after 30 ns and stabilized this “open” configuration. During the 200 ns simulation window, the phosphate ion was not able to diffuse spontaneously from its binding pocket, even after the observed opening of the WPD loop.

A control simulation in which the phosphate ion was removed showed lower fluctuations in the loop containing the WPD motif (0.1 nm on average, compared with 0.2 nm on average when the phosphate was present). Overall, the configuration (Figure 3F, pink color) did not show significant differences compared with the crystal structure.

### 2.4. Secondary Binding Site

**Figure 4:**
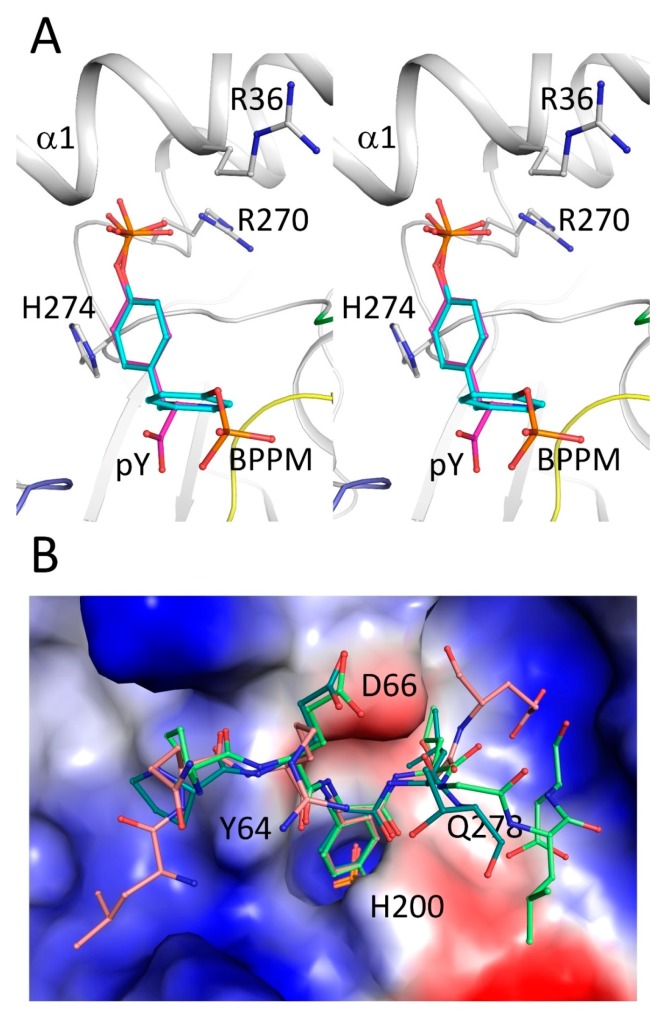
Substrate binding sites of PTPN12. (**A**) A stereoview showing the second substrate-binding site with a docked bis-(*para*-phosphophenyl) methane (BPPM) molecule (**cyan**) and phosphoryl-tyrosine (**magenta**). The coloring of PTPN12 is the same as that used in Figure 1; (**B**) electrostatic potential surface of the PTPN12 substrate-binding site with superimposed, phosphorylated HER2 peptides (side chains shown). The backbones of the HER2 pY1248, pY1196, and pY1112 peptides are shown in green, dark green, and pink, respectively. Important residues in PTPN12 are labeled on the electrostatic potential surface. Figures were prepared using PyMOL (DeLano Scientific LLC, South San Francisco, CA, USA).

A secondary phosphate binding site was observed in a number of PTPs [17,31]. PTPN12 has a “PTP1B-like” pocket (secondary binding site), featured by residues Arg36 and Arg270 (Arg24 and Arg254 in PTP1B) [17,31]. These two basic residues are conserved and capable of interacting with pTyr. The “second-site loop” [17] in our PTPN12 structure formed part of helix α1, which showed a spatial conflict with the superimposed bis-(*para*-phosphophenyl) methane (BPPM) (in PTP1B, PDB ID 1AAX), unless a conformational change was induced upon substrate binding (Figure 4A).

### 2.5. Substrate Recognition

Previously, it was reported that both PTPN18 and PTPN12 can dephosphorylate HER2 [16,28]. The PTPN18/phospho-HER2 peptide structures (PDB IDs 4GFU, 4GFV, and 4NND) [28] were superimposed onto the PTPN12 structure by superposition of the catalytic domains. In the PTPN18 structures, the pTyrs from the HER2 peptides inserted into the active site, forming hydrogen bonds and salt bridges with surrounding residues, including Asp64 (Asp66 in PTPN12) and phosphate-interacting residues in the P-loop. The phenyl rings of the pTyrs are stacked between Tyr62 and Gln276 (Tyr64 and Gln278 in PTPN12), and these crucial residues are all conserved. Specifically, HER2 L1249 can form hydrogen bonds with PTPN18 Gln276 (PTPN12 Gln278), based on the docked structure. Asp1252 of the HER2 pTyr1248 peptide forms a salt bridge with PTPN18 Arg198. That residue is substituted by His200 in the PTPN12 WPD loop. For the pTyr1196 peptide, the backbones of several C-terminal residues form hydrogen bonds with PTPN18 Arg198 (PTPN12 His200). Thus, PTPN12 His200 may play an important role in substrate selectivity (Figure 4B). The HER2 pTyr1112 peptide adopts an extended structure, forming seven backbone hydrogen bonds with PTPN18. In the N-terminus of the pTyr1112 peptide, no side-chain atoms are involved in hydrophilic interactions. All of these conserved and non-conserved residues may reflect the substrate selectivity of PTPs in the NT4 subfamily.

### 2.6. Analysis of Tumor-Derived Mutations

**Figure 5:**
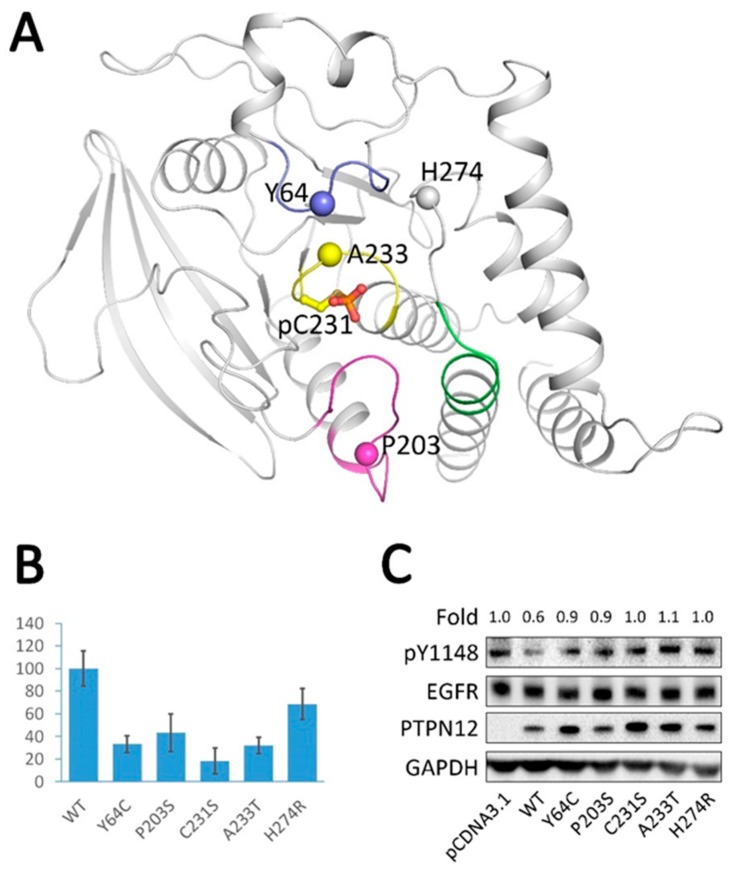
Analysis of tumor-derived mutations in PTPN12. (**A**) Tumor-derived mutation sites are shown as spheres in the model of the PTPN12 catalytic domain. The coloring of the PTPN12 structural elements is the same as in Figure 1. This figure was prepared using PyMOL (DeLano Scientific LLC, South San Francisco, CA, USA); (**B**) comparison of the in vitro catalytic activities between wild type PTPN12 and its variants. All error bars represent the standard deviation from three independent experiments. This figure was prepared using Excel; (**C**) MCF-7 cells were transfected with either wild type or mutant PTPN12 for 24 h, and then treated with 100 ng/mL EGF for 10 min. epidermal growth factor receptor (EGFR) autophosphorylation levels at Tyr1148 and protein-expression levels were measured by Western blot analysis, as indicated. The samples were derived from the same experiment and Western blots were processed in parallel.

Previous findings [16] suggested that PTPN12 suppresses the growth and metastasis of human Triple negative breast cancer (TNBC) cells and that such function is lost by nonsynonymous mutations. Tyr1148 residue of EGFR showed the strongest differential phosphorylation in response to PTPN12 depletion. PTPN12 may also be involved in other types of tumorigenesis [32,33]. We searched the publicly available Catalogue of Somatic Mutations in Cancer (COSMIC) database (http://cancer.sanger.ac.uk/cosmic) of 29337 human primary tumors and found 133 PTPN12 mutations. Based on our crystal structure, we identified four residues in the active site of the protein, which should be crucial for the activity of PTPN12: (1) Tyr64, which interacts with the phenyl rings of substrate peptides; (2) Pro203, which is located in the WPD loop; (3) Ala233, which is located in the P-loop nearby the catalytic residue Cys231; and (4) His274, which is substituted by Met258 in PTP1B and is localized in the secondary substrate-binding pocket [31]. We designed and generated a mutation for each of the mentioned residues (Y64C, P203S, A233T, and H274R) and tested their activity in vitro and in vivo, together with wild type PTPN12 and C231S (Figure 5A). All purified mutants showed lower activity than the wild type PTPN12 catalytic domain in vitro (Figure 5B). To further confirm their activities in vivo, Hela cells were transfected with the plasmids expressing wild type or mutants of the PTPN12 catalytic domain. In contrast to wild type PTPN12, the mutants showed marginal inhibition of EGFR Tyr1148 phosphorylation, even though expression of the mutants was obviously higher than the wild type protein (Figure 5C). Since PTPN12 is a frequently inactivated tumor suppressor in several kinds of cancers [16], our results indicate that PTPN12 inactivation by the selected mutations may play important roles in tumorigenesis in patients.

## 3. Discussion

We solved the crystal structure of the PTPN12 catalytic domain. The catalytic Cys231 in the P-loop acts as a nucleophile, transiently accepting a phosphate ion during the reaction. In our PTPN12 structure, we observed that Cys231 exhibits a dual conformation, putatively corresponding to phosphorylated and dephosphorylated states. The orientation of the phosphorylated Cys231, as well as the conformation of the WPD loop, was consistent with substrate-bound PTPN18 structures. In further MD simulations, using a starting configuration mimicking the release of the phosphate ion, we observed a re-orientation of Cys231 side chain by about 100°, which was consistent with the conformation of dephosphorylated Cys231 in the crystal structure. These results indicate that the phosphorylated Cys231 represents the phosphate-enzyme intermediate in the dephosphorylation reaction. Moreover, the catalytic Cys231 might undergo repositioning to an optimal orientation for the next round of the reaction; such reorientation could result from interaction with the phosphate ion and may be necessary for eviction thereof.

This PTPN12 structure adopted conserved PTP domain folding and shares a number of conserved residues with other family members in the active pocket and ligand binding sites. Considering that some PTPs share common substrates with various activities, our structure together with previously reported PTP structures provides a structural basis for such substrate selectivity. However, the conservation of PTP proteins makes it difficult to develop a highly selective inhibitor. While comparing the structures of PTPN12 and PTPN22, which share conserved inhibitor-interacting residues but have different IC_50_ values with the same inhibitors, it might be possible to develop highly selective inhibitors for specific PTP proteins.

## 4. Materials and Methods

### 4.1. Cloning, Expression, and Purification of Recombinant PTPN12

The catalytic domain of human PTPN12 (1–309) was cloned into the pET-28a expression vector and sequenced. The protein was expressed in *Escherichia coli* BL21(DE3) cells. Cultures were grown at 16 °C overnight after induction with 0.1 mM isopropyl-thio-β-d-galactopyranoside. The harvested cells were resuspended in a buffer comprising 40 mM 2-(4-Morpholino)ethanesulfonic acid (MES) (pH 6.5), 300 mM NaCl, 20 mM imidazole, and 5% *v*/*v* glycerol. The cells were lysed with an EmulsiFlex high-pressure homogenizer and then centrifuged at 15,000 rpm for 30 min. The protein was purified from the lysate supernatant via Ni–nitrilotriacetic acid (NTA) affinity chromatography with standard protocols. The eluted protein was subsequently subjected to size-exclusion chromatography using a Superdex-200 column (GE Healthcare, Pittsburgh, PA, USA) and further purified by HiTrap Heparin HP (GE Healthcare, Pittsburgh, PA, USA). The purity and integrity of the protein was determined by SDS–PAGE. Plasmids encoding mutant PTPN12 variants were generated from the vector containing the wild type enzyme, using the QuikChange Site-Directed Mutagenesis Kit (Agilent, Palo Alto, CA, USA), and the sequences of the PTPN12 variants were confirmed by DNA sequencing. The mutant enzymes were expressed and purified following the same procedures as used for the wild type enzyme.

### 4.2. Crystallization and Diffraction Data Collection

The purified PTPN12 protein was concentrated using a 10,000 Da ultrafiltration membrane (Millipore, Billerica, MA, USA) to 7–9 mg/mL in a solution containing 20 mM MES (pH 6.5). Crystallization experiments were performed at 16 °C using the hanging-drop, vapor-diffusion method. The 1 µL protein solution was mixed with an equal volume of reservoir solution and equilibrated against 0.2 mL of reservoir solution. The initial crystals were grown using commercial screening kits and optimized in a solution containing 0.04 M potassium phosphate monobasic, 16% *w*/*v* polyethylene glycol (PEG) 8000, and 20% glycerol. The crystals were soaked in a crystallization buffer containing 30% PEG 8000 as a cryoprotectant prior to X-ray data collection. X-ray diffraction data were collected at 100 K using an ADSC Quantum 315r detector on beamline BL-5A at the Photon Factory (Tsukuba, Japan) and processed using HKL-2000(HKL Research, Inc., Charlottesville, VA, USA) [34].

### 4.3. Structure Determinations and Refinement

The structure of the PTPN12 catalytic domain was initially solved by the molecular replacement method [35], using the crystal structure of native PTPN22 (Lyp) as a search model (PDB ID 2QCJ). Structure refinement was performed using Refmac5 (Research Complex at Harwell (RCaH) STFC Rutherford Appleton Laboratory, Didcot, Oxfordshire, UK) [36], and model building was facilitated with the program Coot (Medical Research Council (MRC) Laboratory of Molecular Biology (LMB), Cambridge, Cambridgeshire, UK) [37]. PyMOL (DeLano Scientific LLC, South San Francisco, CA, USA) was used for the depiction of structures. The statistics of data collection and structure determination are summarized in Table 1.

### 4.4. Parametrization of Phosphoryl–Cysteine for Molecular Dynamics Simulations

The generalized Amber force field (GAFF) [30] was used to model the phosphorylated Cysteine residue. The Antechamber and Tleap modules in AMBERTools17 suite of programs was employed to generate the initial parameter topology files. After that, the Acpype script was used to convert the parameter topology file to Gromacs format. The restrained electrostatic potential (RESP) charge was determined from quantum chemical calculation at the HF/6-31G (d) level using Gaussian09 package. Prior to calculating RESP charge, we optimized the geometry of the phosphorylated Cysteine molecule at the B3LYP/6-311++G (d, p) level. All the quantum chemical calculations were performed using the solvation model based on density (SMD) implicit solvation model, in consideration of the solvation effect of the water solution.

### 4.5. Molecular Dynamics Simulations

Molecular dynamics (MD) simulations were performed starting from the atomistic coordinates obtained for the crystal structure of the protein the phosphate ion. Simulation was performed using Gromacs 4.6.7 package (version 4.6.7, University of Groningen, Holland, The Netherlands) [38], following the protocol used for the equilibration of other globular proteins (see for example [39]). In brief, after adding hydrogen atoms, the protein model underwent a short energy minimization in vacuo, inserted in a cubic box, and solvated with full atom transferable intermolecular potential with 3 points (TIP3P) water and Cl^−^ and K^+^ ions at a concentration of ~150 mM in order to mimic a physiological ionic strength. Overall, the system contained approximately 73,000 atoms. We initially minimized the energy and then performed equilibrium MD under periodic boundary conditions (the unitary cell chosen was a cube whose sides measured 9 nm) in the constant number of atoms, temperature and volume (NTV) ensemble. Simulations were performed using the Amber03 force field [40]. Parameters and topology for the phosphate ion were generated using the antechamber [41], following the same procedure of previous works [42]. MD trajectory was followed for 200 ns. The temperature during the simulation was held constant at 300 K using the Berendsen thermostat [43]. Fast smooth Particle-Mesh Ewald summation [44] was used for long-range electrostatic interactions, with a cutoff of 1.0 nm for the direct interactions. The Gromacs (University of Groningen, Holland, The Netherlands) [38] and VMD [45] software packages (Beckman Institute for Advanced Science and Technology, University of Illinois, Urbana, IL, USA) were used for statistical analysis.

### 4.6. Phosphatase Activity Assay

Phosphatase assays were performed using a phosphatase assay (PA) Kit (G Biosciences, St. Louis, MO, USA), according to the manufacturer’s recommended protocol. The phosphatase activities of the purified PTPN12 catalytic domain (wild type and mutants) were measured in a 96-well plate. The reaction was performed in a total volume of 150 µL, containing 6 µg of wild type or mutant protein, 44 µL PA assay buffer, and 50 µL PA substrate (pNPP). Assays were initiated by adding enzyme to the reaction mixtures and terminated by adding 50 µL 3N sodium hydroxide after 15 min. The enzyme activity was monitored by measuring the absorbance at 405 nm. The phosphatase activity of PTPN12 was calculated according to the following equation:

Enzyme activity (nmol/(min/μg)) = (OD_405nm_ × V)/(ε × T × L × E), where

Kcat (min^−1^) = (enzyme activity × Mw)/1000

OD_405nm_ = mean absorbance of sample − mean absorbance of blank

V = reaction volume (μL)

ε = extinction coefficient of p-nitrophenol (17.8 mM^−1^cm^−1^)

T = Incubation time (min)

L = path length of light (cm)

E = enzyme (μg)

All assays were performed at 25 °C.

### 4.7. Cell Culture, Transfection, and Treatment

HeLa cells were cultured in a humidified atmosphere in the presence of 5% CO_2_ and 95% air at 37 °C. The cells were transiently transfected with plasmids using the Lipofectamine 2000 reagent (Invitrogen, Carlsbad, CA, USA) according to the manufacturer’s instructions. For Epidermal Growth Factor (EGF) stimulation, the cells were incubated in 100 ng/mL EGF for 10 min, followed by cell lysis.

### 4.8. Accession Code

Coordinates of the structure of the wild type PTPN12 catalytic domain were deposited in the RCSB Protein Data Bank with accession code 5HDE (www.rcsb.org).

## Figures and Tables

**Table 1:** Data collection and structure refinement statistics.

Data Collection	Data Statistics
space group	C2
Cell parameters	
*a*, *b*, *c* (Å); β°	136.01, 40.97, 75.69; 116.63
Resolution (Å)	50.00–1.62 (1.65–1.62) ^a^
Redundancy	6.1 (5.3) ^a^
Completeness (%)	99.9 (99.2) ^a^
*R*_merge_^b^	9.6 (66.2) ^a^
Average *I*/σ *(I)*	37.7 (3.0) ^a^
**Refinement**	
*R*_work_(%)/*R*_free_(%) ^c^	16.3/21.2
Protein atoms (phosphate/water)	2517 (5/354)
RMSD bond length (Å)	0.012
RMSD angle (°)	1.146
Average B-factor	22.70
Protein	21.00
PO_4_	24.42
pCys	16.88
water	34.75
**Ramachandran plot**	
Allowed (%)	99.6
Generously allowed (%)	0.4
Disallowed (%)	0.0

^a^ Numbers in parentheses correspond to the highest resolution shell. ^b^ *R*_merge_ = ∑*i*|*Ii* − ‹*I*›|/∑‹*I*›, where *Ii* is an individual intensity measurement and ‹*I*› is the average intensity for all reflections *i*. ^c^ *R*_work_/*R*_free_ = ∑‖*F*_o_| − |*F*_c_‖/∑|*F*_o_|, where *F*_o_ and *F*_c_ are the observed and calculated structure factors, respectively.
